# Antioxidants in Male Infertility—If We Want to Get This Right We Need to Take the Bull by the Horns: A Pilot Study

**DOI:** 10.3390/antiox12101805

**Published:** 2023-09-27

**Authors:** Usha Punjabi, Ilse Goovaerts, Kris Peeters, Diane De Neubourg

**Affiliations:** 1 Centre for Reproductive Medicine, Antwerp University Hospital, 2650 Edegem, Belgium; ilse.goovaerts@uza.be (I.G.); kris.peeters@uza.be (K.P.); diane.deneubourg@uza.be (D.D.N.); 2 Department of Reproductive Medicine, Antwerp Surgical Training, Anatomy and Research Centre (ASTARC), Faculty of Medicine and Health Sciences, University of Antwerp, 2000 Antwerpen, Belgium

**Keywords:** sperm DNA oxidation, 8-hydroxy-2′-deoxyguanosine, sperm DNA fragmentation, semen parameters, oxidative stress

## Abstract

Antioxidant therapy should be reserved for infertile patients who actually exhibit signs of oxidative stress (OS). Nevertheless, there is no consensus regarding the measure of the primary endpoint and the assay that should be used. The formation of 8-hydroxy-2′-deoxyguanosine (8-OHdG), an early marker of sperm DNA oxidation (SDO), was analyzed using flow cytometry, in men at a University hospital setup for infertility treatment. Similar to conventional semen parameters, 8-OHdG assay was validated on fresh semen samples to reduce the variability of results. SDO was associated with semen volume, sperm concentration, leucocytes and round cells, but not with age, body mass index, sperm DNA fragmentation (SDF) or OS. Whether the semen samples were normal or subnormal according to the WHO criteria, the expression of 8-OHdG was not different. Receiver operating characteristic curve analysis could discriminate two independent populations. Both SDF and SDO were independently expressed. A high SDF did not reveal a high SDO and vice versa. The thresholds for SDO have been established, but vary with the techniques used. The methodology for SDO needs to be further validated and optimized on a larger clinically defined patient population before the outcome measure is fit to monitor antioxidant therapy in male infertility.

## 1. Introduction

Approximately one out of five couples are affected by infertility, which is a worldwide health problem, with male factor infertility contributing to almost half of all cases [1]. Data from a recent meta-analysis [2] suggest a continuing worldwide decline in semen quality at an accelerated pace. There is thus an urgent need to define the causes and taking appropriate actions to prevent the further disruption of male reproductive health [2]. Oxidative stress (OS) is a significant cause of male infertility, and allegedly contributes to the pathology observed in 30–80% of all cases [3,4]. OS occurs when the production of reactive oxygen species (ROS) exceeds natural antioxidant defenses, leading to cellular injury. Several situations have been proven to be associated with the increased production of ROS, such as obesity, varicocele, aging, infection, unhealthy lifestyle-related factors and environmental factors [3,5]. However, ROS are a double-edged sword; on the one hand, they are beneficial for some physiological activities, and on the other hand, when in excess, ROS are detrimental to cell structures, function and survival [6,7]. OS mechanisms affecting sperm function are attributed to peroxidative damage to the sperm axoneme and the depletion of intracellular ATP levels, followed by the generation of 4-hydroxynonenal and malondialdehyde due to the oxidation of lipid membrane components and fragmentation of both nuclear and mitochondrial DNA [8]. Antioxidants are protective against ROS. Perhaps unsurprisingly, antioxidant supplements as potential treatments for male infertility have been investigated in all combinations and doses [9,10,11,12]. Nevertheless, overall the evidence is inconclusive based on serious risk of bias. Increasing evidence also suggests that the irrational administration of oral antioxidants may lead to the disruption of the balance between the oxidative and reductive state, with an immediate effect on sperm physiology [13,14,15]. Without doubt, giving rise to the well-established and undeniable antioxidant paradox, and rendering the whole supplementation procedure a critical challenge [15].

While some clinicians are prescribing antioxidant therapy indiscriminately, the question arises of how to identify the group which needs an antioxidant treatment? If OS is the cause of male infertility, then we need to reserve antioxidant therapy for infertile patients who actually exhibit signs of OS. Studies concerning antioxidant treatment in male infertility have suffered from many flaws such as inappropriate patient selection, inappropriate choice of antioxidant(s), inappropriate dose administration and the pertinence of the endpoints monitored [16]. Various semen parameters have been used as surrogate markers to indicate the effectiveness of antioxidants.

Sperm DNA fragmentation (SDF) is often considered a read-out of OS, but is, in fact, only partly attributable to OS, as it also results from unresolved meiotic breaks, incomplete apoptosis or mechanical breaks during spermiogenesis [17]. However, depending on the OS intensity, the damage to the sperm nucleus can range from the simple oxidation of bases (guanosine and adenosine being the most sensitive bases) to DNA fragmentation, with the generation of abasic sites and DNA–protein cross-linking occurring, as in between oxidative events [18]. According to Lettieri et al. [19], Sperm Nuclear Basic Proteins (SNBP) may be involved in oxidative DNA damage. Histones and protamines contribute to compacting the sperm DNA and thus protecting it from oxidative damage. However, in certain stressful conditions, possible functional alterations of SNBP properties may occur, reversing their canonical protective role and resulting in oxidative DNA damage [20].

The mechanisms of oxidative DNA damage can also cause genetic alterations, resulting in diseases such as cancer and neurodegenerative syndromes [11], as well as some features of aging. Due to a lack of a functional DNA repair system, the sperm cells are unable to correct DNA base alterations [17]. Consequently, during fertilization, the oocyte is presented with unresolved base residues, which the cell has limited capacity to address. As a result, highly mutagenic lesions will persist, increasing the risk of de novo mutations during embryo development [21].

DNA damage, measured via sperm chromatin structure (SCSA), Comet and TUNEL assays, has been highlighted by various Cochrane updates [9,10,11,12] that antioxidant treatment is far from effective for male infertility. Moreover, the absence of SDF should not be interpreted as the absence of SDO, as DNA fragmentation alone does not provide a complete picture of genomic damage. While measuring DNA oxidation could be another option, reports on the effects of antioxidants on oxidative DNA damage are rather scarce.

Bearing in mind that OS represents a relevant clinical issue to male gametes, the present investigation was set in motion to implement the formation of 8-hydroxy-2′-deoxyguanosine (8-OHdG), an early biomarker of DNA oxidation. This pilot study was undertaken to evaluate the most recent, objective technique in a clinical andrology laboratory, working according to strict WHO standards. In addition, the measurement of oxidative-reductive potential was implemented to give an idea of the OS in the sample, and SDF was measured using the TUNEL assay to study whether fragmentation occurred simultaneously with DNA oxidation.

## 2. Materials and Methods

### 2.1. Study Protocol

This was a prospective, observational study approved by the Ethical Commission of the Antwerp University Hospital and the University of Antwerp. Sperm DNA oxidation (SDO) was conducted between March–May 2023 in an infertile population, approved on 21 December 2020 (Belgian registration no: 20/45/591).

### 2.2. Participants

The study population comprised a cohort of patients undergoing infertility diagnosis or intra-uterine insemination treatment at the Centre for Reproductive Medicine, Antwerp University Hospital, Belgium. Male partners between 18 and 65 years of age, with a normal semen analysis/mild male subfertility (defined as one or more abnormal diagnostic semen parameter with a total progressive motile sperm count above 5 million according to WHO [22]) were eligible for the study. Conversely, subjects with clinical pathologies (such as varicocele, testicular infection, prostatitis or testicular torsion), congenital anomalies, inherited genetic abnormalities, azoospermia (no spermatozoa), cryptozoospermia (few hidden spermatozoa) and extreme oligo-, asthenozoospermia were excluded. All subjects had given written informed consent for participation. For the inclusion criteria, it was of priority to observe that the eventual diagnosis and treatment of the patient concerned was not jeopardized by the additional tests carried out on the sperm samples.

### 2.3. Procedures and Interventions

All participants filled in a clinical male fertility diagnosis questionnaire covering lifestyle parameters and personal medical history (Supplemental Data included).

#### 2.3.1. Semen Analysis

Semen samples were collected through masturbation at the laboratory, and then analyzed within 60 min of production. Standard semen parameters were analyzed according to WHO 2010 [22] recommendations, with appropriate internal and external quality control measures [23,24,25].

#### 2.3.2. Oxidative Stress (OS)

The Male Infertility Oxidative System (MiOXSYS) was used to measure the sperm oxidation–reduction potential (sORP), the balance between total oxidants and reductants, and, thus, a direct measure of OS in semen samples [26]. The value in millivolts (mV) was normalized by dividing it with sperm concentration to control for differences in cell numbers, and results were presented as mV/M/mL semen. In an initial work (unpublished data) a significant ROC curve (*p* < 0.0001) was generated in sORP levels between normal and subnormal semen samples, producing a cut-off value of 1.94 mV/M/mL.

#### 2.3.3. Sperm DNA Fragmentation (SDF)

Assessment of SDF was performed using the terminal deoxynucleotidyl transferase–mediated deoxyuridine triphosphate nick-end labelling (TUNEL assay) described by Mitchell et al. [27]. Approximately 2 million sperm cells were first decondensed with 2 mM dithiothreitol (DTT, Sigma-Aldrich, Overijse, Belgium) and then fixed in 3.7% formaldehyde (Sigma-Aldrich, Overijse, Belgium). Following permeabilization (100 mg Sodium citrate, 100 µL Triton X–100 in 100 mL dH_2_O), the sperm cells were washed and analyzed using the fluorescein In Situ Cell Death Detection Kit (Roche Diagnostics, Mannheim, Germany) using Accuri C6 flow cytometer (BD Sciences, Erembodegem, Belgium). DNA fragmentation was analyzed in the total sperm sample at a flow rate of 35 µL/min, recording 5000–10,000 events/sample. The positive controls were treated with DNase I (Qiagen, Germany), while for the negative controls all components were included except the terminal deoxynucleotidyl transferase enzyme. The method has been standardized and threshold values of normality (≤13% SDF) established as compared to a fertile cohort [28,29].

#### 2.3.4. Sperm DNA Oxidation (SDO)

The method described by Vorilhon et al. [30] was implemented to detect OS through the presence of 8-OHdG in sperm samples. Briefly, a part of the semen sample (approximately 2M sperm cells) was incubated with decondensation buffer (2 mM DTT, 0.5% Triton X-100 in PBS) for 10 min in the dark, followed by a wash with PBS. Thereafter, the positive control was incubated with 1.5% H_2_O_2_ (Sigma-Aldrich, Overijse, Belgium) for 1 h at room temperature. Subsequently, the cells underwent an additional wash followed by fixation in 3.7% paraformaldehyde for 20 min at 4 °C. After the fixation, the cells were washed and 1.5% normal goat serum (Sigma-Aldrich, Overijse, Belgium) was added to counteract nonspecific antibody binding. After an incubation of 1 h, anti-8-OHdG monoclonal antibody (Novus Biologicals, Centennial, CO, USA) was added with a final dilution of 1:770 (except blank and negative controls) for overnight. Following a wash procedure, the secondary antibody (diluted at a ratio of 1:770), conjugated with Alexa Fluor 488 (goat anti-mouse second antibody, Life Technologies, Thermofisher, Brussels, Belgium), was introduced and allowed to incubate for a duration of 90 min (except blank). After this, the cells were washed one last time and the pellet resuspended in PBS. Just before measurement, 10 μL propidium iodide (PI, Sigma-Aldrich, Overijse, Belgium) was added to exclude M540 apoptotic bodies. These non-nuclear apoptotic bodies were located at the same position in the FSC/SSC dot plot in flow cytometry as spermatozoa, and would otherwise interfere with sperm 8-OHdG quantification. At least 10,000 sperm cells were analyzed at a flow rate of ±200 events/sec using a BD Accuri C6 FCM. Alexa Fluor 488 and PI fluorescence were measured on excitation by a blue laser at 488 nm and were paired with emission measurements using the Standard Optical Filter 533/30 nm (FL-1) and the Standard Optical Filter 670 nm LP (FL-3), respectively. The positive controls were treated with H_2_O_2_, while for the negative controls all components were included except the anti-8-OHdG antibody. Results were expressed as percentage of 8-OHdG positive spermatozoa and the mean intensity level of fluorescence (MIF).

### 2.4. Statistical Analysis

Statistical analyses were conducted using Medcalc^®^ version 20.027—64-bit (MedCalc Software Bv, Oostende, Belgium). Median, mean, standard deviation (SD) and ranges were reported for the patient characteristics, semen parameters and OS parameters. Logarithmic transformation was applied to obtain normal distribution (Kolmogorov-Smirnov test) in semen variables where necessary, followed by back transformation. Spearman correlation was used to test the associations between patient characteristics, semen variables and OS parameters. 8-OHdG and MIF affecting age, BMI, semen parameters, SDF and sORP, were tested using univariate linear regression analysis. For multivariate analysis, the significant variables in the univariate analyses were used in a multiple linear regression analysis. To assess differences in continuous variables between two groups, the Mann–Whitney test was used in case the data were not normally distributed. Differences in continuous variables between three or more groups were assessed using the Kruskal–Wallis tests. If significant, the groups were compared pairwise using a post hoc test. Comparisons of the data distributions between SDF and SDO were conducted through receiver operating characteristic (ROC) curve analysis. For all statistical tests, differences with a *p* value <0.05 were considered significant.

## 3. Results

In this pilot study, semen parameters were assessed in 78 samples. The descriptive characteristics of the different variables are given in Table 1. SDO assessment was prioritized above SDF and sORP when limited by semen volume/sperm concentration, which accounts for the low numbers. Sperm morphology was available for diagnostic samples and was not carried out on IUI samples included in the study. When round cell concentration exceeded 1 M/mL, the peroxidase-positive differentiation of leukocytes was advocated.

### 3.1. Sperm DNA Oxidation Correlation Analyses

Sperm DNA oxidation expressed as percentage of 8-OHdG-positive sperm or as MIF were not significantly associated with age, BMI, SDF and sORP (Table 2).

Classifying the weight status by BMI, 28 (54.9%) had a normal weight (18.5–25 kg/m^2^); 14 (27.5%) were overweight (25–30 kg/m^2^) and 9 (17.6%) were obese (>30 kg/m^2^). Neither % 8-OHdG (*p* = 0.359) nor MIF (*p* = 0.398) showed any significance between the different categories.

**Figure 1:**
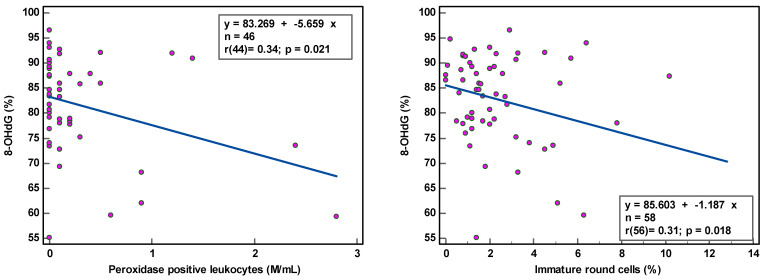
Correlation of sperm DNA oxidation expressed as percentage of 8-hydroxy-2′-deoxyguanosine-positive spermatozoa (8-OHdG %) with semen parameters.

**Figure 2:**
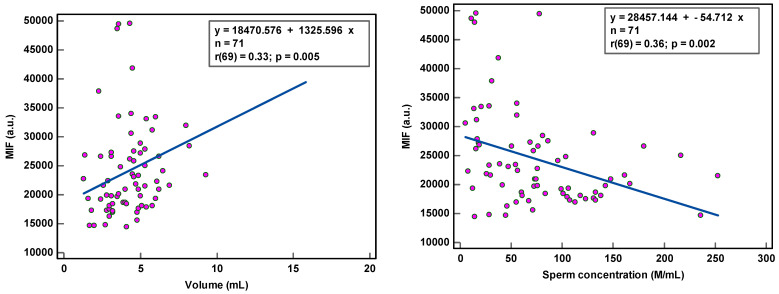

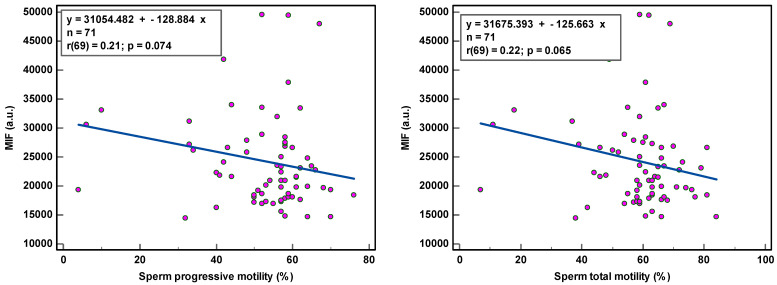
Correlation of sperm DNA oxidation expressed as mean intensity fluorescence (MIF) in arbitrary units (a.u.) with semen parameters.

8-OHdG-positive spermatozoa were found to be weakly associated with peroxidase-positive leucocytes (r(44) = 0.34, *p* < 0.021) and round cells (r(56) = 0.31, *p* = 0.018) (Table 2; Figure 1), while MIF was weakly correlated with semen volume (r(69) = 0.33, *p* = 0.005) and sperm concentration (r(69) = 0.36, *p* = 0.002) (Table 2; Figure 2). With multivariate analyses, only MIF remained associated with semen volume and sperm concentration (Table 3).

The correlation coefficients (r) of 0.31–0.36 reveal weak associations between semen parameters and SOD. Although statistically significant, due to the low number of samples analyzed, we are more cautious in deciding that there is a relationship between these variables. The coefficient of determination (R²), a measure of effect size, ranged between 9.61 and 12.96%, revealing that semen variables would account for only ±11% of the variance in SOD, as 89% of the variability in SOD would be unrelated to semen parameters.

The observation of the dots scattering regarding the correlation line supports a cautious interpretation of the data gathered in this study. The small sample size renders the obtained correlation unstable. Moreover, semen samples with low sperm concentration and low sperm motility and high round cell and high peroxidase-positive leukocyte concentrations were not sufficiently represented, which could affect the strength of the correlation. A few outliers in the higher values could distort correlations considerably.

### 3.2. Sperm DNA Oxidation in Normal and Subnormal Samples

**Figure 3:**
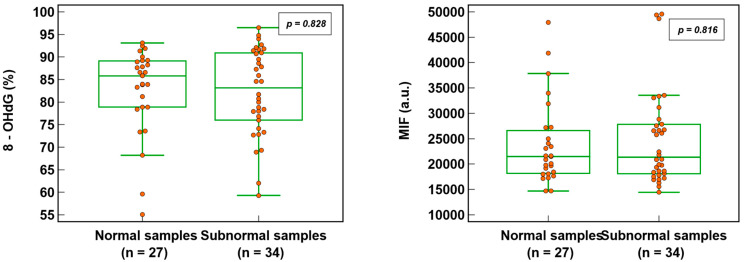
SDO in normal and subnormal semen samples expressed as percentage of 8-hydroxy-2′-deoxyguanosine-positive spermatozoa (8-OHdG) and mean intensity fluorescence (MIF) in arbitrary units (a.u.).

Sperm DNA oxidation, expressed as % 8-OHdG (82.6 ± 9.6% vs. 82.2 ± 9.6%; *p* = 0.828) or MIF (23,848.7 ± 8268.6% vs. 24,894.1 ± 9417.7; *p* = 0.816), was not significantly different (Figure 3) whether the semen samples were normal or subnormal according to the WHO criteria [22]. Out of 63 patients (where all three semen parameters were analyzed), 27 (42.9%) had normal semen values and 36 (57.1%) were subnormal (with one or more abnormalities).

### 3.3. ROC Analysis and Threshold Values for Sperm DNA Oxidation

**Figure 4:**
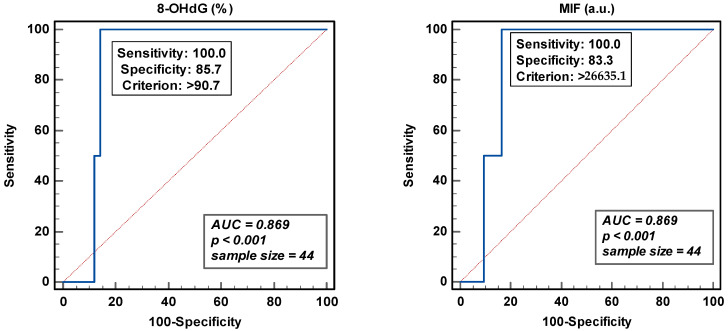
Receiver operating curve analyses for percentage of 8-hydroxy-2′-deoxyguanosine-positive spermatozoa (8-OHdG) and mean intensity fluorescence (MIF) in arbitrary units (a.u.).

The area under the curve (AUC) gives an indication of the ability to discriminate two independent populations. Total sperm count was used to obtain the Youden threshold value of >26635.1 for the MIF and the peroxidase-positive leukocytes for 8-OHdG (>90.7%) (Figure 4). In either case, even though the sensitivity was excellent, the specificity of the analysis was around 83.0 and 85.0%. The low sample size and the discontinuous distribution of the samples analyzed could affect this trade-off between sensitivity and specificity.

**Figure 5:**
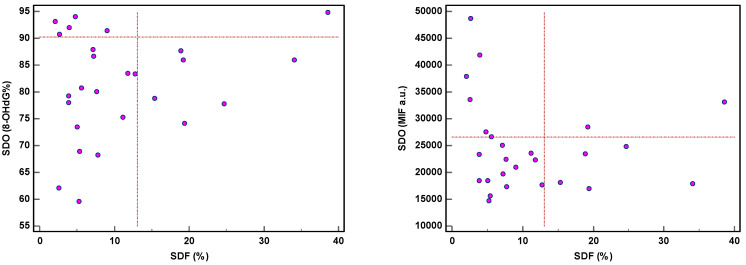
Expression of sperm DNA fragmentation (SDF) and sperm DNA oxidation (SDO) as percentage of 8-hydroxy-2′-deoxyguanosine-positive spermatozoa (8-OHdG%) and mean intensity fluorescence (MIF) in arbitrary units (a.u.).

The threshold of 8-OHdG was revealed in 14/71 (19.7%) patients and MIF in 20/71 (28.2%), while SDF and sORP were high in 7/26 (26.9%) and 5/29 (17.2%) patients, respectively [28]. Moreover, both SDF and SDO were independently expressed (Figure 5), except in one or two samples. High SDF did not reveal a high SDO, whether expressed as 8-OHdG (%) or as MIF. The same applied in case of high SDO.

## 4. Discussion

As OS can come from many quarters, it is hard to predict exactly who is likely to be suffering from OS. In animal models, there is evidence that antioxidant therapy is extremely efficient against OS [21]. However, in humans, as Aitken [16] puts it, we are racing to assess the value of antioxidant therapy before developing the necessary protocol to detect OS and monitor its intensity in the face of antioxidant therapy. The literature reveals that earlier studies quantifying 8-OHdG using HPLC coupled with electrochemical detection [31,32,33] have been criticized as affected by the possible spontaneous formation of 8-OHdG during the step of the extraction/digestion of sperm DNA [34,35,36]. Direct assays, which are ELISA-based, have also not served as a ‘gold standard’ for the measurement of 8-OHdG [37,38,39]. Conversely, these different assays have reported a diversity of baseline values. Later on, techniques employed antibodies or binding proteins [40,41,42] combined with microscopy. However, this is time-consuming, subjective and requires a long learning curve [30]. Here, we have undertaken a primary step to implement a robust protocol to measure oxidative DNA damage.

Although the methodology is objective using anti 8-OHdG antibody and flow cytometry [30], our study reveals a relatively high percentage of 8-OHdG-positive sperm cells (81.7% vs. 66.6%) and MIF (22,597 vs. 937) identified compared to that observed by Vorilhon et al. [30]. These relatively high values, compared with previous observations [42,43,44,45], were attributed to the DNA decondensation pretreatment via DTT, which increases antibody access to DNA 8-OHdG sites [30]. However, the limited number of samples analyzed in our study and that by Vorilhon [29] could be a serious limitation.

Significant correlations have been reported between SDO and isolated semen parameters, including sperm concentration [30,32,37,46,47], sperm motility [30,39,42,44,48,49] or sperm morphology [30,45]. Our results demonstrated a negative correlation with sperm concentration, suggesting that high levels of SDO are associated with the condition of oligozoospermia. Kodama [32] speculates that in these patients, spermatozoa with extensive DNA damage were generated and absorbed during the spermatogenetic process, resulting in an oligozoospermic condition. A similar trend was observed, but not a significant one, with sperm motility and morphology. SDO revealed weak correlations with semen parameters, which suggests that the causal relationship between the two variables is ambiguous. The small sample size, the discontinuous distribution of data, and outliers could make correlations obtained in some situations quite unreliable. Nonetheless, semen parameters provide fundamental information on which clinicians base their initial diagnosis. Our results reveal that semen variables would account for only ±11% of the variance in SOD; the remaining 89% of the variability in SOD would be endogenous- or exogenous-related ROS production unrelated to semen parameters, which does not allow for the exclusion of a random effect. In addition, 8-OHdG an OS are not supported as biological markers for stress-induced infertility. Moreover, no differences in the levels of SDO were observed between normal and subnormal samples, suggesting that sperm DNA oxidation in normozoospermic samples may be one of the factors related to unexplained male infertility [50,51,52,53]. Evidently, just as sperm DNA fragmentation [29,54], oxidative DNA damage may be considered an independent attribute of semen quality for all infertility patients, detecting problems not seen with semen analysis alone.

SDO was related to high polymorphonuclear neutrophil cells, as observed by the positive correlation obtained by Vorilhon et al. [30] in their study group including 14 leukocytopsermic samples. With only four peroxidase-positive samples in our study population, we observed a significant negative correlation between the percentage of 8-OHdG-positive sperm cells and peroxidase-positive leukocytes.

The clinical thresholds for SDO have been established but vary with the techniques used [30,31,32,33,34,35]. Vorilhon [30], with a moderate AUC, has proposed optimal cut-off points for SDO. In this study, the ROC analysis gave us higher threshold values with a good AUC and a high sensitivity and specificity. High sensitivity is important for a screening/diagnostic test so that it can be offered to a larger population, while specificity becomes critical if a test is to be offered as a predictive marker of a defined endpoint. Vorilhon et al. [30] observed a significant correlation between sperm DNA oxidation and BMI. A higher MIF was observed with overweight patients compared to the normal weight group. We could not confirm this finding, probably due to the low numbers analyzed or the patient selection bias. Moreover, diet could have been an important modifiable determinant here, which was not considered. According to Ferramosca and Zara [55], ‘a western diet’ is considered a risk factor for metabolic diseases, atherosclerosis and cancer, as well as male infertility, due to the high intake of industrially processed foods compared to the ‘Mediterranean diet’, which is composed of vegetables, fruits and seafood [55].

Although baseline/cut-off values have been reported for 8-OHdG, discriminating threshold values of normality, as compared to a fertile cohort, should be established to validate this molecule as a biomarker for oxidative stress damage. Even though fertile populations are enriched with good quality samples expressing low levels of DNA damage, transitory oxidative stress may occur even in the absence of an infertility diagnosis.

In our previous work [54], we have shown that one out of three patients with a high SDF can benefit from an oral supplementation. On the contrary, one in five showed an increase in SDF after supplementation. Whether this imbalance observed was due to reductive stress could not be attributed to the formulation of the nutritional supplementation used [13,56]. While a pro-oxidative situation may lead to SDF, it should not be considered as synonymous with SDO alone. On the other hand, an equally if not more relevant observation is that the absence of SDF at a level considered pathological should not be interpreted as the absence of SDO. In fact, it has been shown that the oxidation of the bases of sperm DNA is more frequent than SDF. In a panel of men from infertile couples, 2 to 3 out of 10 had a level of SDF considered as pathological, but 6 to 7 out of 10 had a moderately to highly oxidized sperm nucleus [30,57]. Since SDO does not explain all the SDF, and since the absence of fragmentation cannot be an assurance of the absence of oxidation, it is clear that the two parameters (SDF and SDO) must be evaluated to properly qualify the state of the sperm nucleus and develop an appropriate therapeutic strategy [58]. Our results have substantiated this observation on a small number of samples.

In conclusion, our findings demonstrate that 8-OHdG formation can be implemented as an early biomarker of DNA oxidation. combining immunofluorescence with flow cytometry. The strength of this article lies in the choice of the methodology used to detect SDO after the decondensation of the compact chromatin. But, the technique has its limitations; it is labor-intensive and not low-cost. While this test shows promise for clinical andrology use, the conclusion inferred from this pilot study should be taken with caution. The low number of samples analyzed and the discontinuous distribution of the semen variables produced weak correlations of SOD with semen parameters, implying no causality. The technique needs to be further optimized and validated on a substantial number of observations, including samples with both high and low semen variables, before being implemented for clinical purposes. The lack of an optimal commercially available kit might become a treat for 8-OHdG as a bio-marker of OS. Moreover, fertile populations are distinctly different from subfertile men in that they are enriched with good-quality samples expressing low levels of DNA damage. However, transitory oxidative stress may occur in the absence of an infertility diagnosis. The methodology provides opportunities to determine threshold values of normality using a fertile cohort, and implementing the method in different populations of men attending an infertility clinic should unravel the effect of oxidative damage of ejaculated spermatozoa in human reproduction. As stated in the recent guidelines of the American Society for Reproductive Medicine, the clinical utility will only be confirmed after the standardization of methodology and clinically applicable threshold values, which would be reached by the same criteria [59].

## Figures and Tables

**Table 1:** Descriptive statistics of all participants.

Variables	Samples Analyzed	Median	Mean ± SD (Range)
Patient characteristics			
Male age at diagnosis (years)	77	35.0	35.3 * ± 7.1 (25.0–60.0)
BMI (kg/m^2^)	51	24.1	25.4 * ± 4.2 (18.8–36.7)
Non-smokers	42 (77.8%)		
Smokers	12 (22.2%)		
Semen variables			
Semen volume (mL)	78	4.3	4.1 * ± 2.1 (1.3–15.8)
Sperm concentration (M/mL)	78	67.7	54.1 * ± 54.6 (5.4–252.9)
Total sperm count (M/ejaculate)	78	238.3	219.6 * ± 276.1 (23.8–1340.4)
Sperm progressive motility (%)	78	57.0	51.1 ± 15.0 (4.0–76.0)
Sperm total motility (%)	78	61.0	57.6 ± 15.6 (7.0–84.0)
Sperm morphology (%)	68	3.0	3.8 ± 2.7 (0.0–16.0)
Peroxidase-positive leukocytes (M/mL)	50	0.1	0.3 ± 0.6 (0.0–2.8)
Immature round cells (M/mL)	63	1.8	2.6 ± 2.4 (0.0–12.8)
OS variables			
SDF (%)	30	7.5	8.3 * ± 9.6 (2.1–38.6)
sORP (mV/M/mL)	29	0.8	0.8 * ± 1.2 (0.1–4.6)
8-OHdG spermatozoa (%)	76	84.0	81.7 ± 9.0 (55.1–96.5)
MIF (a.u.)	76	21,797.9	22,597.9 * ± 8397.9 (10,749.5–49,551.1)

* Backtransformed after logarithmic transformation. BMI = body mass index; SDF = sperm DNA fragmentation; OS = oxidative stress; sORP = oxidation–reduction potential; 8-OHdG = 8-hydroxy-2′-deoxyguanosine-positive spermatozoa; MIF = mean intensity of fluorescence using arbitrary units (a.u.).

**Table 2:** Evaluation of parameters affecting sperm DNA oxidation by Univariate Linear Logistic Regression.

Parameters		8-OHdG		MIF	
	Samples Used	Coefficient (SE)	*p* Value	Coefficient (SE)	*p* Value
Age (years)	71	−0.014 (0.153)	0.926	−92.174 (143.587)	0.522
BMI (kg/m^2^)	51	−0.105 (0.315)	0.741	−292.387 (249.293)	0.247
Semen volume (mL)	71	0.466 (0.509)	0.363	1255.734 (455.853)	0.007
Sperm concentration (M/mL)	71	−0.009 (0.019)	0.627	−54.020 (16.758)	0.002
Total sperm count (M/ejaculate)	71	0.003 (0.004)	0.474	−5.943 (3.458)	0.089
Sperm progressive motility (%)	71	−0.081 (0.074)	0.279	−126.659 (68.710)	0.069
Sperm total motility (%)	71	−0.061 (0.071)	0.394	−129.507 (65.170)	0.051
Sperm morphology (%)	61	−0.695 (0.440)	0.120	−747.101 (394.853)	0.063
Peroxidase-positive leukocytes (M/mL)	46	−5.397 (2.303)	0.023	−1153.933 (2385.824)	0.631
Immature round cells (M/mL)	58	−1.222 (0.478)	0.013	314.839 (478.497)	0.513
SDF (%)	26	0.246 (0.182)	0.187	−89.629 (170.611)	0.604
sORP (mV/M/mL)	29	1.709 (1.579)	0.289	2089.054 (1147.332)	0.079

SE = standard error; SDF = sperm DNA fragmentation; sORP = oxidation–reduction potential; 8-OHdG = 8-hydroxy-2′-deoxyguanosine-positive spermatozoa; MIF = mean intensity of fluorescence using arbitrary units (a.u.).

**Table 3:** Evaluation of parameters affecting sperm DNA oxidation using multivariate analyses.

Parameters	Samples Used	MIF	
		Coefficient (SE)	*p* Value
Semen volume (mL)	71	1188.453 (428.432)	0.007
Sperm concentration (M/mL)	71	−50.051 (16.203)	0.003

SE = standard error; MIF = mean intensity of fluorescence using arbitrary units.

## Data Availability

The data presented in this study are available from the corresponding author on a reasonable request.

## Supplementary Materials

The following supporting information can be downloaded at: https://www.mdpi.com/article/10.3390/antiox12101805/s1, Supplemental Data: Clinical male fertility diagnosis questionnaire.
