# Supplementary material for: Antioxidants in Male Infertility—If We Want to Get This Right We Need to Take the Bull by the Horns: A Pilot Study
Source: Antioxidants (Basel). 2023 Sep 27;12(10):1805. doi: 10.3390/antiox12101805 (PMC10603832; doi:10.3390/antiox12101805)
Supplement: Supplementary file 1 [file antioxidants-12-01805-s001.zip › antioxidants-2533912-supplementary.pdf]

UZA / Drie Eikenstraat 655 / 2650 Edegem
Tel +32 3 821 30 00 / www.uza.be / BE0874.619.603

Centrum voor Reproductieve Geneeskunde
Drie Eikenstraat 655
2650 Edegem

QUESTIONNAIRE MAN

Personal data

Date when the questionnaire was filled out:………/………/…………
Name: ……………………………………………………………………………………………
Name of your partner: ……………………………………………………………………………
Address:……………………………………………………………………………………………
Date of birth:…………………………………………… Date of Birth of your Partner:………/………/………
Telephone number :…………………………………… Mobile Phone : ……………………………………
Email:………………………………………………………………………………………………
Current profession:…………………………………………………………………………………

Information about your doctor(s)

○ No referring doctor

General practitioner (Family doctor)
Name: ……………………………………………
Address:…………………………………………
……………………………………………………
Tel:……………………………………………

Gynaecologist/urologist/endocrinologist
Name:……………………………………………
Address: …………………………………………
……………………………………………………
Tel:…………………………Fax:…………………………
Hospital:…………………………………………

Describe your request for help to our Center

……………………………………………………………………………………………………
……………………………………………………………………………………………………
……………………………………………………………………………………………………

Since when do you have childwish? ……………………………………………………………

What is the duration of your relationschip? ……………………………………………………

Are you married or do you live together? ………………………………………………………

Bloodgroup:……………………………………

Do you have children/miscarriages in your current relationship? ○ no ○ yes, how many? ……………

Do you have children/miscarriages in a previous relationship? ○ no ○ yes, how many? ……………

Family history

Are there any relatives in your family with:

	No	Yes	If Yes, please explain:
Congenital anomalies:	○	○	………………………………
Heriditairy disease	○	○	………………………………
Fertility problems	○	○	………………………………
Psychological problems (depression, schizofrenia….):	○	○	………………………………
Other causes not mentioned:	○	○	………………………………

UZA / Drie Eikenstraat 655 / 2650 Edegem
Tel +32 3 821 30 00 / www.uza.be / BE0874.619.603

Centrum voor Reproductieve Geneeskunde
Drie Eikenstraat 655
2650 Edegem

Personal history

Did you ever suffer from a severe disease?
○ no ○ yes If Yes, please specify the name of the disease and the name of the doctor who is following up on you :
....

Did you ever suffer from a depression or did you take any antidepressants?
○ no ○ Yes If Yes, please specify when this oocurred and if you are still in follow up by a physician:
....

Did you ever experience problems at the testicle(s) or penis?
○ no ○ yes If Yes, please specify:..

Did you ever have problems in having or mainitaining an erection? ○ no ○ yes

Did you ever have problems with ejaculation? ○ no ○ yes

Did you ever have surgery? ○ no ○ yes If Yes, please specify the year and the type of surgery: ....
....

Did you ever have surgery for a varicocoele (tortuous veins on the testicle) / surgery for cryptorchidia (undescended testicle(s)/ infection/ prostatitis/ torsion of the testicled/phimosis? ○ no ○ yes (If yes, circle)

Are you allergic to medication (antibiotics,...), latex or desinfectants?
○ no ○ yes If yes, please specify:..

Do you take medication? ○ no ○ yes
If yes, please specify the name and dose of the medication: ....

Lifestyl man

Do you smoke? ○ no ○ yes If yes, how many sigarettes do you smoke in a day?..........................

Do you drink alcohol? ○ no ○ occasionally ○ yes If yes, how many units of alcohol per day/week? ....

Do you or did you ever use soft or hard drugs ○ no ○ yes If yes, please specify:..............................

Are you in contact with toxical substances? ○ no ○ yes If yes, please specify:..

Do you work in specific conditions (extreme heat eg)? ○ no ○ yes

Weight: ....kg
Length: ....cm

Information on previous fertility treatments

Did you ever have any treatment for subfertilty? ○ no ○ yes
If yes, who was your doctor? ....

What was the cause of the subfertility?
....
